# What does an enabling environment for infant and young child nutrition look like at implementation level? Perspectives from a multi-stakeholder process in the Breede Valley Sub-District, Western Cape, South Africa

**DOI:** 10.1186/s12889-018-5165-7

**Published:** 2018-02-13

**Authors:** L. M. Du Plessis, M. H. McLachlan, S. E. Drimie

**Affiliations:** Division of Human Nutrition, Faculty of Medicine and Health Sciences, Stellenbosch University, PO Box 241, CAPE TOWN, 8000 South Africa

**Keywords:** Enabling environment, Infant and young child nutrition, Knowledge and evidence, Politics and governance, Capacity and resources, People-centred approach

## Abstract

**Background:**

Breede Valley is a sub-district of the Cape Winelands district, Western Cape Province, South Africa. The administrative capital of the district is situated in the semi-rural town Worcester. Findings of a baseline survey in Worcester revealed poor infant feeding practices and childhood under- and overnutrition, with particular concern over high levels of stunting and low dietary diversity. Maternal overweight and obesity was high. These characteristics made the site suitable to study multi-sectoral arrangements for infant and young child nutrition (IYCN). The purpose of this study was to explore elements of an enabling environment with key stakeholders aimed at improving IYCN at implementation level.

**Methods:**

Focus group discussions and interviews were conducted with representatives from two vulnerable communities; local and district government; higher education institutions; business; and the media in the Breede Valley. Audio recordings were transcribed and data were analysed with the Atlas.TI software programme.

**Results:**

The participants viewed knowledge and evidence about the first 1000 days of life as important to address IYCN. The impact of early, optimal nutrition on health and intellectual development resonated with them. The IYCN narrative in the Breede Valley could therefore be framed around nutrition’s development impact in a well-structured advocacy campaign. Participants felt that capacity and resources were constrained by many competing agendas spreading public resources thinly, leaving limited scope for promotion and prevention activities. “People” were viewed as a resource, and building partnerships and relationships, could bridge some shortfalls in capacity. Conversations about politics and governance elicited strong opinions about what should be done through direct intervention, policy formulation and legislation. A lead government agency could not be identified for taking the IYCN agenda forward, due to its complexity. Participants proposed it should be referred to a local, informal, inter-governmental body where directors and senior managers meet to address issues of cross-cutting importance.

**Conclusion:**

The study illustrated that knowledge and evidence; politics and governance; and capacity and resources, elements of the international definition of an enabling environment, also apply at implementation level. In addition, our findings indicated that a people-centred approach is critical in shaping the enabling environment at this level.

## Background

Over a decade ago, Heaver [1] pointed to governance challenges lying at the heart of slow progress, when he highlighted limited commitment of countries to address infant and young child nutrition (IYCN) [1]. Today the governance for IYCN encompasses the complex interactions between public and private entities with the aim of reaching shared goals and actions to improve IYCN [2], which is receiving much more attention on the global agenda [3]. With commitments publicly stated, the focus has now shifted to creating the conditions, or the “enabling environment” [4], to strengthen capacity and action to turn commitments into results, particularly at implementation level [2–7]. This has been no easy undertaking.

Gillespie et al. [4] systematically reviewed the nutrition-relevant policy process and governance literature to define an enabling environment for improved nutrition [4]. The authors proposed that a trio of interlinked elements; *knowledge and evidence, politics and governance, and capacity and resources*; are needed to shape and maintain momentum for addressing malnutrition and for translation of this momentum into outcomes [4].

The research reported here formed part of a multi-stakeholder engagement process at implementation level in the Breede Valley, Western Cape Province, South Africa. The Breede Valley is a sub-district of the Cape Winelands district in the Western Cape Province, South Africa. The administrative capital of the district is situated in the semi-rural town Worcester, about a hundred kilometres from Cape Town, one of the largest towns in the Western Cape and the centre of the province’s interior region. This town and district also form part of Stellenbosch University’s rural service and community engagement area. It is home to a satellite campus of the university, referred to as the “Worcester Campus” where staff and students from the Faculty of Medicine and Health Sciences are involved in community engagement and internships. Findings of a baseline survey in two vulnerable communities in Worcester, published elsewhere, revealed poor IYCN [8]. Poor feeding practices and childhood under- and overnutrition, notably poor breastfeeding practices, poor complementary feeding practices and high levels of stunting (almost a third of children aged 0–23 months) were prevalent. Dietary diversity of young children was low. Overweight and obesity affected two thirds of women of childbearing age. When the IYCF practices and child anthropometric profile were considered parallel to the maternal anthropometric profile, it was clear that nutritional practices in the first 1000 days of life placed the future development, growth and health of children in the Breede Valley in serious jeopardy [8]. These characteristics made the sub-district a suitable site to study multi-sectoral arrangements for IYCN.

The multi-stakeholder engagement research project included different phases, i.e.: systematic stakeholder identification and categorisation, exploratory interviews with selected key stakeholders, followed by a multi-stakeholder engagement workshop. Insights were gathered on stakeholders’ perspectives about IYCN, their commitment and capacity to address the situation in the Breede Valley sub-district as well as relationships and powers among stakeholders in the domain of IYCN. The last phase of this research, reported here, set out to critically reflect on the research process and explore local conditions conducive for action (i.e. the enabling environment) aimed at IYCN at implementation level.

The conceptual question and sub-questions set for this specific phase of the research were:

- What does an enabling environment for IYCN look like at implementation level?
  - ○ What types of issue framing approaches and narratives grab the attention for IYCN in different contexts?
  - ○ Which elements are crucial for an enabling environment at implementation level to create momentum for change in IYCN?, and,
  - ○ Who should be leading the process of change for IYCN at implementation level?

## Methods

Four focus group discussions and two interviews (in cases where participants were not available for the focus group discussions) were held with a total of 19 stakeholders. These included representatives from two vulnerable communities (grandmother, crèche owners, sport club organizer, community development/volunteer workers, politician, unemployed mother and a teenager); local municipality (Cape Winelands) and district government departments (Department of Health, Education and Social Development); higher education institutions (Stellenbosch University satellite campus and Boland College); the local business forum and the media (local radio station) in the Breede Valley (Table 1). The participants were selected through a stakeholder identification and differentiation process from the earlier phases of the research.

**Table 1:** Stakeholders from different organisations represented in focus group discussions and interviews

○ Group 1 – Community members: grandmother/crèche owner, community volunteer worker, teenager (*n* = 3)
○ Group 2 – Community members: crèche owner, sport club organizer, community development worker, politician, unemployed mother (*n* = 5)
○ Group 3 – Representatives from Cape Winelands District Government (Departments of Health, Higher Education institutions (Stellenbosch University & Boland College); The local Business Forum, Local Media (n = 5 mid-level manangers)
○ Group 4: Representatives from Cape Winelands District Municipality (Community Development and Planning) and Cape Winelands District Government (Departments of Health x 2 and Social Development) (*n* = 4 mid- and senior level managers)
○ Interview 1: Cape Winelands District Government (Department of Social Development – social worker)
○ Interview 2: Cape Winelands District Government (Department of Education – newly appointed senior manager)

The predominant languages in this area are Afrikaans, English and isiXhosa. Most residents speak and understand at least one or a combination of these languages. Participants indicated which language/s they were comfortable with, before commencement of the interviews and focus group discussions. The first author is bilingual in Afrikaans and English and conducted all the interviews and focus group discussions in one of these two preferred language. An isiXhosa translator was available and her services was used upon request for one group member in Group 1. Focus group discussions and interviews lasted for about an hour. All focus group discussions and interviews were conducted in a private, quiet environment.

Ethics approval for this research project was granted by the Human Research Ethics Committee (HREC), Faculty of Medicine and Health Sciences, Stellenbosch University (Reference nr: S13/03/043). Permission for employees of government to take part in the focus group discussions/interviews was requested and received from the applicable provincial and/or district departments/sectors/line-managers, including from the Departments of Health (DOH), Education (DOE), Social Development (DSD) and Community Development and Planning (CDP). Written informed consent was requested from all participants and separate written consent was requested to record the focus group discussions/interviews. Confidentiality was ensured by keeping transcriptions password protected, removing all personal identification from records and by keeping the list of interviewees separate from interview data. Anonymity was ensured by referring only to codes and themes in the results reporting.

The introduction to the focus group discussions and interviews included a reflection on what is known about IYCN in the Breede Valley [8] and how the research process had unfolded during the different phases of the research. This reflection set the scene for the opening question: “*How should we tell the story about IYCN in the Breede Valley?”* to gauge possible ideas around framing of the narrative.

Following this discussion, elements of an enabling environment to address the IYCN situation at sub-district level, were explored. The international definition developed for an enabling environment, was used to initiate the discussion; i.e.:

*“political and policy processes that build and sustain momentum for the effective implementation of actions that reduce undernutrition”* [4]

The terms “political and policy” as well as “enabling environment” were discussed in simpler language in cases where this terminology was not well understood by groups or individuals. The following question was then explored: “*How would you describe an enabling/supportive environment to create momentum for change in IYCN in the Breede Valley*?”

Vocabulary, descriptions or discussions involving the three linked elements of an enabling environment (i.e.: *knowledge and evidence; politics and governance; capacity and resources*) [4], were carefully recorded.

The interviewer did not mention any of these elements, but the interviewer paid specific attention to additional elements that were mentioned as critical at implementation level and probed to elicit a clear articulation of these elements. A final question: “*Who should be leading the agenda for IYCN at implementation level?”* was also explored. The interviewer encouraged any additional and last perspectives and opinions before closing the discussions.

The FGDs and interviews were structured around three broad themes: i) telling the story of IYCN; ii) elements that describe an enabling environment and iii) stakeholder/s to lead the IYCN agenda. These themes will be reported in relation to the three linked elements included in the definition of an enabling environment: 1) *knowledge and evidence; 2) politics and governance and 3) capacity and resources* [4] to gain insight about the “look and feel” of such an environment for IYCN at implementation level.

### Data analysis

The audio recordings of the interviews and focus group discussions were sent to professional transcribing services. Recordings were transcribed in English with simultaneous translation from the interview language, if necessary. After the interviews were transcribed, the first author performed quality control on the data by listening to the recordings while reading the transcripts to ensure that information was captured accurately.

Transcriptions of focus group discussions and interviews were separately entered into the “Atlas.ti” software programme (Atlas.ti Qualitative Data Analysis, 2015) [9] and assigned to a unique hermeneutic unit (file) to enable labelling segments of text (quotes) with a string of words/phrases (codes). The first author established these codes after careful reading and re-reading of the text. Once all the transcriptions were coded, the codes were grouped into “families” (main themes) which are larger groups of codes for easier handling in reporting the data.

The data was not verified by a second person, but the co-authors were familiar with the data from all the phases of the research project and assisted with the interpretation and integration of the codes and main themes.

## Results

### Knowledge and evidence

Reflecting on the research process, it was acknowledged that local research by Stellenbosch University in the sub-district has contributed to building the knowledge-base of, among other, the profile of selected facets of the first 1000 days of life in the Breede Valley. Knowing about the local situation was described as a starting point to finding collective solutions for the IYCN problems faced. The University’s social responsibility to do relevant research and to disseminate this knowledge in understandable language to communities who participate in research was mentioned.

“…the researchers bring the evidence to the table in our area, so we have that privilege.” (FGD 3: Participant 03).

Applying “the importance of the first 1000 days” catch phrase seemed to be an efficient way of approaching the narrative of IYCN in the Breede Valley. Participants expressed the realisation of the knowledge of the problems and the need for evidence-based interventions would have to be fed back into the training of professionals in the sub-district.

Although there has been progress on growing the local information and research base, comments about the absence of advocacy, limited communication on the topic and the huge need for IYCN information was reiterated. Concern was expressed that the full impact of poor nutrition within the first 1000 days of life is not widely known, specifically referring to developmental delays and poor schooling outcomes, which are not explicitly connected back to nutrition.

Deliberating the various factors in a mother’s immediate environment as well as her distant environment which influence her ability and choices to feed her children highlighted the need and importance of cross-cutting and standardised, focussed messages on the first 1000 days of life. The audience for IYCN was considered to be extensive and it was mentioned that “everyone” is in need of the messages to work towards solutions.

“The clinic sisters and the doctors and mommy and those who help me and so, everyone, even the employer or those who provide jobs to them, they should be part of the solution.” (FGD 1: Participant 02).

It was acknowledged that interventions take time to be successful and should therefore be paced, but should remain focussed in order to clear the many misconceptions that are still evident in communities around the topic of IYCN.

Apart from a focus on cognitive development and schooling performance, not much more was offered in terms of content needed for advocacy messages. This was considered to be left to experts in communication. Rather, the discussions steered towards who should be communicating the case for IYCN in the Breede Valley and on which platforms.

All the different government departments, including DOH, DOE, DSD and CDP were cited as stakeholders who should be telling the story of IYCN. This would include providing information and education on the importance of nutrition within the first 1000 days of life and how optimal IYCN during this time-period impacts the life course. Conversely, there was recognition of the overburdened government system with its multiple focus areas and the realisation that it will take time to consolidate agendas and actions for IYCN. Nonetheless, this would have to be addressed as it was a priority to establish multi-sector collaboration. Therefore, a designated organisation, possibly an NGO which receives funding from government, was proposed as means of communicating messages and dealing with the problem of IYCN in a holistic manner. The NGO “FASFacts” (*FASFacts*, 2015),^1^ has done work in the sub-district and was cited as a model which could be copied for IYCN. “FASFacts” aimed to educate the general public on the effect of alcohol consumption during pregnancy on the unborn baby, as well as the influence that people with fetal alcohol syndrome have on each individual as well as the broader community.

The use of NGOs to assist in IYCN advocacy with a non-judgemental approach was deemed important in order to ensure buy-in from community members in particular. Bearing in mind the time it takes to do advocacy and for a campaign to reach its target, it was suggested that follow-up “reminders” should be built in at different points of contact with mothers / caregivers and children and between government sectors. In this way the message about the importance of the IYCN can be developed and built into people’s frames of reference. Although it was felt that IYCN should be strongly emphasised within a consolidated campaign, it was stated that even small beginnings can ripple out positively to the community.

“…we should make sure that as the kid moves along, which ever channel that is, that there's a message reminder or a message carrier at every point. We need to enable people at all levels to carry the message. If we've agreed that this (*a focus on IYCN*) is the universal message that needs to go into the system, then we must mature the messages all over the place.” (Interview 02).

It was stated that training curricula of students from healthcare professions needed to address IYCN in the context of prevention of disease and promotion of good health. These students were mentioned as partners who could spread the messages. The media were also included as a partner who should be communicating the messages. Further, the business sector was identified as having a social responsibility to assist in telling the IYCN story.

A number of places and spaces were suggested to this effect. It was stated that the story could be told anywhere in venues where people meet; including clinics, or venues close to clinics; churches; occupational health services in the workplaces; crèches and in the home. For fathers, more unorthodox venues were listed, including at shebeens,^2^ in prison, and at sports events. Special gatherings could also be organised specifically to address IYCN. Breastfeeding week on the international and national health calendars was seen to provide a good focus on IYCF. A limitation was its one week duration. It was felt that special information days and classes should be organised for pregnant and lactating mothers.

IYCN was perceived as a topic that lends itself to visual stimulation. Traditional material including pamphlets; posters; and billboards could be supplemented by electronic media, including cellular telephones; the internet; and Facebook, were mentioned as information carriers for IYCN. It was, however, acknowledged that literacy is problematic as is access to technology. An innovative suggestion was made that taxis can be used as vehicles, literally and figuratively, to carry IYCN messages in communities.

### Politics and governance

Politics at community level were described as more than just about political parties, but also included the “political atmosphere in the community”. Reference was made to issues people perceived to be important in their communities and their needs for the space where they live and work, which ultimately implicate the involvement of people.

There was acknowledgement that distant political decisions, particularly those in Pretoria where central government was situated, impact the local/implementation level. Although this is the case, it was also felt that politicians in communities, the ward councillors, should be using their power to create jobs to make a real impact in local communities.

It was stated that government has a responsibility towards its citizens and therefore the community should have a voice. It was also felt that government should, in particular instances, use their power to take action in the form of policy and legislation and enforce certain issues, including those pertaining to IYCN. Early childhood development (ECD) centres were mentioned as one such specific case where government should enforce attendance to ensure improved nutrition and care for vulnerable infants and young children.

“If there is a policy in place, then - take for example all the children from six months to five years old, they should be in a crèche or else the ‘Allpay’ (*government grant to unemployed parents*) will be taken away.” (FGD 1: Participant 01).

Such a tough measure mentioned to enforce ECD attendance, with the aim of improving IYCN, in part highlight the level of intervention needed. It also implies that government should dramatically increase the focus on IYCN.

The role of policy and legislation in addressing maternity leave in the formal and informal sectors was also highlighted as an avenue that can be used to support IYCN.

“How do we make it possible with maternity leave to speak exclusive breastfeeding for six months, what does our maternity leave policy say?” (FGD 3: Participant 02).

“I think that most farm workers get maternity leave but they are not paid. But the question obviously is who must pay? If generally the farm is under pressure and receive little subsidy from the government, it is perhaps the State that must pay the maternity leave.” (FGD 3: Participant 03).

The lack of conversations on issues of mutual importance, in general, between government departments was expressed as a reason why there is difficulty in successfully addressing cross-cutting issues.

“…we don’t have common conversations; we have silo conversations and that’s part of why we struggle in my view, anyway.” (Interview 02).

Government departments explained the difficulty of their work by referring to the fact that they do not have control over the social determinants of health. Nonetheless, certain solutions were posed, with one specific opportunity, created by law, in the Intergovernmental Relations (IGR) Framework Act (Act No. 13 of 2007) [10]. This act encourages decision makers to engage around important issues at a local level. This might pose a real opportunity to extend the reach and power of planned interventions.

There was a strong feeling that funds for IYCN initiatives should be dedicated from national and provincial government, and not from other smaller entities, to ensure sustainability of interventions. Also, it was said that the decisions on dedicated budget changes to support IYCN interventions should be made at a higher level i.e. provincial and national.

Elevating the Integrated Nutrition Programme in the hierarchy of Department of Health, from an integrated programme to a more prominent programme, could raise the profile of nutrition and support budget shifting, among other, in order to have an impact on IYCN. Comments were also made in relation to accountability structures for decisions and interventions either directed through a mandate or as an issue that needs careful integration with performance management.

“But somewhere the decision must be made that we have the mandate that we can go back if it doesn’t work and say: ‘listen they are dragging their feet’ or they must say: ‘no, where is Education, where is Health?’” (FGD 4: Participant 01)

### Capacity and resources

In the domain of capacity and resources, strong impressions arose of government officials being overwhelmed with high workloads as they faced many different focus areas as well as a lack of resources. It was therefore felt that the organisational capacity to make the case for IYCN was weak in the Breede Valley. This partly explained the lack of advocacy, information and communication for IYCN. A further explanation was that the curative focus on the burden of chronic diseases, overshadow health promotion and prevention activities in terms of emphasis and resources. There was reluctance to take up an issue that was not aligned with different stakeholder departments’ budgets, targets and objectives and thus a difference in priority setting. This was offered as an explanation of why there may be no clear lead for IYCN.

A need for champions to fly the flag for IYCN was expressed and it was acknowledged that “champions for a cause” can make a big impact. It was also mentioned that champions are overburdened with many pressing issues since they are often the same people involved in many different causes. A dedicated post for a champion to drive such a process was not seen to be viable from DOH’s point of view, due to the integrated way in which implementation is operationalized. Pooling of resources from all the departments to fund such a post was also not seen as feasible since no structure currently exists to bring such a suggestion to fruition. The proposition of an outside partner (NGO) to focus specifically on IYCN with dedicated funds from government was restated as a possible bridging measure. It was thought, however, that an NGO should not be the sole driver of the cause, since a government department will still need to be the link back to the funding entity.

Perspectives about who, with specific reference to a government department, should be leading the IYCN agenda, were varied. There was reference made to the fact that IYCN should be strongly linked to behaviour change, and since there is no custodian for behaviour change, it is difficult to say who should actually be leading the agenda. DOH was highlighted by DSD and DOE as the department who has IYCN as part of its core business and who should be taking the lead on advocating for IYCN. But DOH felt that problems are already manifested by the time children reach them. The other departments felt the same about their situations, in that it is “too late” for them to intervene when the problem presents itself at their door, confirming the prevailing curative as opposed to preventative approach followed by government departments.

There was general consensus that a specific forum, the Liaison group should be the driving committee where departments can deliberate working together on IYCN issues. This unique forum has been in existence in the Breede Valley since 2009 and is focussed on intergovernmental cooperation. The Liaison group comprises the directors and senior managers of DOH, DOE, DSD and CDP. They meet quarterly and discuss issues of transversal importance. This group is regarded as a hub of strategic capacity where issues of importance can be tabled, decisions can be made and accountability exerted. The directors represented at this group were described as leaders with passion who want to do the right thing. They believe in their workforce and inspire them through their leadership. Therefore, they know they can make decisions and their staff will keep working on the momentum for actions since their sense of purpose fuels dedication. It was acknowledged that relationship building is difficult to do. But trust, partnerships and relationships were seen as important forms of capacity within the government sector in particular, that could bridge the lack of other resources.

Resources in communities were said to be very scarce and families rely on one another for daily living and support. Parents often have to make difficult decisions about the care of their young children.

“If I work on a farm and I am called; the truck stops – this is what happens in Avian Park and De Doorns. ‘You can harvest today’. Then I leave that child with anybody because I can bring home R150 tonight. That’s the reality.” (FGD 4: Participant 01).

It was said that there are some parents who do not care about their children’s wellbeing, tying in with comments about a breakdown in family values. A need for incentives in communities were therefore expressed, e.g. for mothers to breastfeed and to change their current behaviour. Since family and community values were felt to be jeopardised and family units have disintegrated, religious institutions were mentioned as a resource in communities which could rekindle these values.

### Additional perspectives on the enabling environment at implementation level

In response to the question about possible elements lacking from the definition of an enabling environment, as applicable to the implementation level, important responses emerged. Some participants responded that “policy to practice” was seen as difficult, especially since the reality of problems in society is in competition with IYCN, including the social determinants of health. Besides, not all stakeholders see a direct alignment with the first 1000 days concept. There are also different environments impacting implementation level, including: the immediate environment (households, communities and service providers), but also the distant environment (provincial, national and global).

“Communities” and “people” were components absent from the global definition and this result in elements of “human behaviour” and “behaviour change” also being vague. These elements were felt to be of particular importance at implementation level, since people and communities need to respond to policies, legislation and interventions differently compared to, among other, policy makers, legislators and researchers in other levels in the hierarchy of a decentralised system.

“It is people who let the things happen.” (FGD 4: Participant 01)

“Partnerships” and “relationships” might be implicit to the global definition, but it was felt that these two elements were particularly important at implementation level, as to warrant explicit citation. Motivation for the highlighting of these elements was mentioned in the fact that building partnerships and relationships, as well as sustaining it, is difficult, but necessary. A good working environment and retention of staff was seen as one means of sustaining relationships and partnerships. Furthermore, it was communicated that people at implementation level adapt to limited resources by working with what is available by taking up an enduring attitude and believing that, although challenges persists, solutions are possible.

Shared values, especially trust, were seen as essential elements in relationship building. Relationships in itself were seen as a resource, which could bridge the shortfall in resources which is often experienced at implementation level. In turn, good working environments that include incentives were seen as enablers for fostering loyalty and positive attitudes towards the work that needs to be done.

## Discussion

The discourse around the importance of the first 1000 days of life, and the knowledge about the effective interventions during this very specific time period in the lifecycle, have been communicated powerfully on a global level [2, 11, 12]. This serves as confirmation of how well-packaged research can bring focus and create momentum for a cause [4]. Framing of the first 1000 days of life within a well-defined focus area has been said to be of specific importance [12]. Countries have approached capturing their own IYCN storyline from different angles, including an emphasis on hunger elimination in Guatemala and Bolivia; undernutrition reduction as an election strategy in Peru; lack of economic growth in India; and agriculture as a means of economic growth and reduction of poverty in Ghana [4, 7]. During discussions in the Breede Valley, stakeholders strongly related to cognitive development and scholastic achievement and the resultant increase in human capital that could be achieved through investment in the 1000 days’ time period. The storyline for IYCN in the Breede Valley could therefore be framed to address this development concern.

The Lancet 2008 Series on Maternal and Child Undernutrition [13] highlighted the lack of cohesion in messages, priorities and funding for the international nutrition community. Five years later in the Lancet 2013 Series on Maternal and Child Nutrition [4], there was consensus that much more consistency and harmonizing was evident in the way different actors and organizations interact at a global level, but it should not be a surprise that there was a lack of advocacy at the local level [14]. The need for a well-planned communication and advocacy strategy was highlighted throughout the stakeholder engagement process in the Breede Valley. A similar point was expressed in a SA report (2014) concerning “Evaluation on nutrition interventions for children from conception to age 5” [15]. An explicit recommendation in this report was that DOH should create a specific, well-defined, and dedicated promotion and communication strategy on nutrition for young children, including a focus on the first 1000 days of life. It was further stated that the proposed strategy should borrow from experience gained from the HIV/AIDS campaign for the country [15]. A unique entity in the Breede Valley that could support such a process at implementation level is the university’s satellite campus. With the community’s participation, the university can build evidence, assist in translating the evidence into understandable messages, and also apply evidence through evidence-based interventions. With the right knowledge, information and interventions, the enabling environment can be strengthened.

Participants offered information, explanations and vocabulary that corresponded with the three linked elements needed for an enabling environment. It can therefore be said that these elements are recognised as important and applicable at the local level as well. Important additional components, not explicit in the international definition, were proposed to be “people”, “community” and “behaviour”. This could collectively be worded as “people-centeredness”. People and not the structures they work in, are said to be most important and need to be appropriately directed through participatory leadership [16].

In addition, elements of specific importance at implementation level, warranting stronger emphasis within the three linked elements, and often referred to as “soft issues” were highlighted as: relationships; partnerships; trust; sustainability of organisational structures; and retention of staff. Relationships and partnerships between government departments, the university, business sector, media and the community were discussed. Trust building between all levels was seen as necessary to keep relationships and partnerships strong. Sustainability in structures and staff was also seen as enablers for cultivating an enabling environment. According to literature, these value-based collaborations consistently lead to successful partnerships [16].

A definition of the enabling environment at implementation level could therefore expand on the international definition, as follows:

“*political, policy* AND PEOPLE-CENTRED *processes that build and sustain momentum for the effective implementation of actions that reduce malnutrition*”

The proposed expansion of this definition could be introduced into international debates and agendas for continued deliberation, research and action to improve IYCN as a global, national and local priority.

Furthermore, in order to pull forces together and address IYCN in a comprehensive and integrated way, there is a need for facilitation of various multi-stakeholder interventions [4]. Different sectors need to be able to see reciprocal advantages of and for their involvement. This in turn could galvanise the political will needed for action [17]. One unresolved issue for the participants in this study was the question on organisational leadership for IYCN at implementation level. The Integrated Nutrition Programme (INP), embedded within the cluster: “Comprehensive health services” of Department of Health in the Western Cape, could be seen as a natural champion for IYCN. However, the low profile, limited resources and therapeutic-focused budget of the INP [15] seemingly limits its current capacity to step up to this challenge. Evidence from other countries reveal that it is not uncommon to question the Nutrition component’s capacity to lead issues of importance for the advancement of nutrition, regardless of the Ministry in which they are positioned [1, 18]. An inconspicuous position, multi-sectoral action required, lack of funds and capacity are cited for the poor ability to lead [15, 18]. Lessons learnt from global and national level research indicate that nutrition champions and strong leadership are key in developing and sustaining political commitment and capacity to ensure action [19, 20]. Strong leadership is also needed at various levels along the continuum of multi-sectoral collaboration [16]. Lack of such capacity as well as training and support for such individuals are barriers to effective and sustained commitment for nutrition [2, 19, 20]. This form of strategic capacity is also fundamental for advancing commitment building, agenda setting, policy formulation and capacity building for operations in nutrition [21]. With only one nutrition leadership course on the African continent (African Leadership Programme), there is a certain need for training institutions, the private sector and government to collaborate on investing in leadership capacity for nutrition [4] including in South Africa. In the case of the Breede Valley, in the absence of a lead government agency, much confidence was expressed in the Liaison group as a forum to collectively take up the IYCN agenda. Referring the IYCN issue to the Liaison group might unlock inter-sectoral ownership, leadership and governance as a key strategy in taking the IYCN agenda forward in this sub-district.

The Liaison group in the Breede Valley is, however, not a legally constituted body, and seems to be unique in its existence at sub-district level in the Western Cape Province and possibly the country. In the absence of such forums elsewhere in the country, a way of taking nutrition issues forward faster at implementation level in SA, could be through creating a broader enabling environment based on the Intergovernmental Relations (IGR) Framework Act (Act No. 13 of 2005) in SA [10]. The Act legislates the establishment of appropriate IGR forums at the three spheres of government, in particular the provincial sphere, vertically tied to the premier’s IGR forum. The premier’s forum must engage actively with various district IGR forums convened by district mayors. District Intergovernmental forums (DIF) are consultative forums to facilitate intergovernmental relations between districts and locals. The primary role of this forum is to discuss national and provincial directives affecting municipalities. This offers a potential infrastructure for multi-sectoral engagement on malnutrition and other issues, as stipulated in the SA National Development Plan (NDP) 2030 [22]. Since ad hoc invitations to attend the intergovernmental forums may be made on the discretion of the chairperson (district mayor), this is an opportunity that warrants investigation for advocacy and action for IYCN. These, however, remain questions that will have to be tested in additional empirical research in follow-on processes to the reported research.

## Limitations

The participants sample size was small. However, the initial group was established through a systematic stakeholder identification process during the first phase of the overall research project. A manageable number of participants had to be chosen based on the facilitation guidelines of the specific stakeholder engagement methodology followed. Therefore, the group could not be too big. Also, in the context of qualitative research, the numbers reached are not relevant. Conclusions drawn are therefore applicable to the research setting and not necessarily generalizable. Important lessons learnt can, however, be shared with stakeholders working at implementation level elsewhere in the province and possible the country.

## Conclusion

This research explored the look and feel of an enabling environment for IYCN at implementation level in the Breede Valley, Western Cape, South Africa. A framing approach that grabbed the attention of stakeholders in the Breede Valley related to cognitive development and scholastic achievement and the resultant increase in human capital that could be achieved through investment in the 1000 days’ time period. The storyline for an advocacy strategy on IYCN in the Breede Valley could therefore be framed to address this development concern.

It was established that the crucial elements of an enabling environment at implementation level depend on knowledge and evidence from the local context; politics and governance; and capacity and resources to create momentum for change in IYCN. In addition, a people-centred approach is critical to move the IYCN agenda forward and for successful interventions at this level.

Stakeholders found it difficult to identify a clear organisational lead to spearhead a change process for IYCN at implementation level. A unique intergovernmental forum, the Liaison group, could potentially galvanise inter-sectoral ownership, leadership and governance for IYCN in this sub-district.

## Footnotes

1 FASfacts uses experiential learning to convey the FAS message in local communities and nationally throughout South Africa. In order to reach these communities they establish satellite operational offices in communities (*FASFacts*, 2015).

2 A shebeen is a pub or bar located in a township that provides alcohol, music and dancing as well as a meeting place for local community members. https://en.m.wikipedia.org/wiki/Shebeen.

## References

[1] Heaver R (2005). Strengthening country commitment to human development: lessons from nutrition.

[2] Nisbett NC, Gillespie S, Haddad L, et al. Why worry about the politics of childhood undernutrition? World Dev. 2014; 10.1016/j.worlddev.2014.06.018.

[3] Mejia Acosta A, Fanzo J (2012). Fighting maternal and child malnutrition: Analysing the political and institutional determinants of delivering a national multisectoral response in six countries. A synthesis paper.

[4] Gillespie S, Haddad L, Mannar V, et al. The politics of reducing malnutrition: building commitment and accelerating progress. Lancet. 2013; 10.1016/S0140-6736(13)60842-9. 10.1016/S0140-6736(13)60842-9 23746781

[5] Chopra M, Pelletier D, Witten C (2009). Assessing countries’ readiness: methodology for in-depth country assessment. 37^th^ session papers.

[6] Haddad L. How can we build an enabling political environment to fight undernutrition? Eur J of Dev Res. 2012; 10.1057/ejdr.2012.45.

[7] International Food Policy Research Institute (2014). Global nutrition report 2014: actions and accountability to accelerate the world’s progress on nutrition.

[8] Du Plessis LM, Herselman MG, McLachlan MH, et al. Selected facets of nutrition during the first 1000 days of life in vulnerable south African communities. S Afr J Child Health. 2016; 10.7196/SAJCH.2016.v10i1.984.

[9] Atlas.ti Qualitative Data Analysis. 2015. http://atlasti.com/. Accessed 27 Sept 2016.

[10] Intergovernmental Relations Framework Act, No. 13 of 2005. Government Gazette. 825(27898). 3 august. Government notice no. 696. Cape Town: Government Printer; 2005.

[11] Black RE, Victora CG, Walker SP, et al. Maternal and child undernutrition and overweight in low-income and middle-income countries. Lancet. 2013; 10.1016/S0140-6736(13)60937-X. 10.1016/S0140-6736(13)60937-X 23746772

[12] Pelletier D, Haider R, Hajeebhoy N, et al. The principles and practices of nutrition advocacy: evidence, experience and the way forward for stunting reduction. Matern Child Nutr. 2013; 10.1111/mcn.12081. 10.1111/mcn.12081 PMC6860839 24074320

[13] Morris SS, Cogill B, Uauy R. For the maternal and child undernutrition study group. Effective international action against undernutrition: why has it proven so difficult and what can be done to accelerate progress? Lancet. 2008; 10.1016/S0140-6736(07)61695-X. 10.1016/S0140-6736(07)61695-X 18206225

[14] Haddad L, Nisbett N, Barnett I (2014). Maharashtra’s child stunting declines: what is driving them? Findings of a multidisciplinary analysis.

[15] Department of Performance Monitoring and Evaluation. Evaluation of Nutrition Interventions for Children from Conception to Age 5. Department of Health, Department of Social Development & Department of Performance Monitoring and Evaluation. The Presidency, Republic of South Africa, South Africa, Pretoria. http://www.nutritionsociety.co.za/attachments/article/76/Summary-Evaluation-of-Nutritional-Interventions-for-Children-from-Conception-to-Age-5-.pdf. 2014. Accessed 27 Sept 2016.

[16] Garrett J, Kadiyala S, Kohli N (2014). Working multisectorally to improve nutrition: global lessons and current status in India. POSHAN policy note 1.

[17] Harris J, Drimie (2012). Toward an integrated approach for addressing malnutrition in Zambia: A literature review and institutional analysis.

[18] Natalicchio M, Garrett J, Mulder-Sibanda M (2009). Carrots and sticks: the political economy of nutrition policy reforms.

[19] Bryce J, Coitinho D, Darnton-Hill I, et al. For the maternal and child undernutrition study group. Maternal and child undernutrition: effective action at national level. Lancet. 2008; 10.1016/S0140-6736(07)61694-8. 10.1016/S0140-6736(07)61694-8 18206224

[20] Shrimpton R, du Plessis L, Delisle H, et al. Public health nutrition capacity: assuring the quality of workforce preparation for scaling up nutrition programmes. Public Health Nutr. 2016; 10.1017/S136898001500378X. 10.1017/S136898001500378X PMC10271189 26857753

[21] Pelletier DL, Menon P, Ngo T, et al. The nutrition policy process: The role of strategic capacity in advancing national nutrition agendas. Food Nutr Bull. 2 Suppl:2011, S59–S69. 10.1177/15648265110322S203 21916115

[22] National Planning Commission. National Development Plan 2030: Our future – make it work. Pretoria: The Presidency. http://www.gov.za/issues/national-development-plan-2030. 2012. Accessed 27 Sept 2016.

